# Digital Health Tool for Preventing Blindness From Diabetic Retinopathy: Protocol for a Qualitative Study

**DOI:** 10.2196/65894

**Published:** 2025-11-12

**Authors:** Akua Frimpong, Alvaro Granados, Thomas Chang, Julia Fu, Serina S Applebaum, Shannan G Moore, Mahima Kaur, Bolatito Adepoju, Vignesh Hari Krishnan, Amanda Levi, Terika McCall, Kristen Harris Nwanyanwu

**Affiliations:** 1 Department of Ophthalmology and Visual Science Yale School of Medicine New Haven, CT United States; 2 Larner College of Medicine at University of Vermont School of Medicine Burlington, VT United States; 3 Kaiser Permanente Bernard J Tyson School of Medicine Pasadena, CA United States; 4 Division of Health Informatics Department of Biostatistics Yale School of Public Health New Haven, CT United States; 5 Department of Biomedical Informatics and Data Science Yale School of Medicine New Haven, CT United States

**Keywords:** diabetes mellitus, DM, diabetic retinopathy, mobile apps, digital health tool, community engagement, preventable blindness, user-centered design, mobile health, mHealth, digital tool, blindness, prevention, qualitative study, community-led

## Abstract

**Background:**

Diabetic retinopathy (DR), a leading cause of preventable blindness among working-age adults, leads to worse health outcomes among Black, Latine, and individuals with lower income in comparison to other ethnic, racial, and socioeconomic groups in the United States.

**Objective:**

We aim to engage community members directly to identify barriers and facilitators of DR screening and co-design a digital health tool that is accessible, user-friendly, and community-responsive.

**Methods:**

We conducted focus groups with individuals from the Greater New Haven, Connecticut area, aged 18 years or older, and diagnosed with diabetes to (1) conduct a comprehensive disease-management needs assessment and (2) inform the development of a community-responsive digital health tool to optimize DR education and to increase access to DR screening in high-risk populations. We transcribed the focus group interviews, used rapid qualitative analysis to generate themes, and completed affinity mapping to identify content and features for a digital health tool for preventing blindness from DR.

**Results:**

We recruited and interviewed 19 individuals (13/19, 68% female; 9/19, 47% Black; 5/19, 26% Hispanic; 2/19, 11% Native American or Alaska Native) for 4 focus groups. Over 94% (18/19) had access to smart devices other than a computer. Participants self-reported mean hemoglobin A_1c_ of 6.77 (SD 1.93). Approximately 58% (11/19) of the participants completed some college. Frequently occurring themes (>30) obtained from coding contextual interviews were desired app attributes such as options for customization, covering topics such as, the mental impact of living with diabetes, informal support (eg, peer support), diabetes education, barriers to controlling blood sugar and maintenance of health, dietary guidance, and notifications. We created high-fidelity wireframes incorporating key features. Next, we will iteratively design a prototype with community feedback.

**Conclusions:**

We anticipate that this study will characterize the experiences of people living with diabetes. Using a community-led approach,we will continue to analyze the data that will generate insights regarding the key features, content, and benefits that are most important for the creation of a digital health tool for DR prevention.

**International Registered Report Identifier (IRRID):**

DERR1-10.2196/65894

## Introduction

Diabetic retinopathy (DR), a leading cause of preventable blindness among working-age adults, represents a significant public health challenge, particularly among racial and ethnic minoritized communities in the United States [1,2]. Despite advances in therapeutic interventions, disparities in screening and treatment outcomes persist, disproportionately affecting non-Hispanic Black, Latine, American Indian, Alaska Native, and lower-resourced communities [3-6]. These groups are less likely to be screened for DR and are more likely to present at advanced stages of the disease. They also face significant barriers to follow-up care, which ultimately leads to a higher prevalence and severity of the condition [4-8].

The underutilization of DR screening services in these high-risk populations highlights a critical gap in our current health care delivery models, which fail to account for the nuanced needs of diverse communities. Moreover, existing health technologies, including mobile health tools, often do not reach or effectively serve marginalized groups, further exacerbating health disparities [9-13]. This inequity stems from a variety of complex factors, including socioeconomic conditions, structural racism, historical underrepresentation in health research, and lack of culturally tailored health interventions [14-17].

Addressing these challenges requires innovative approaches that embrace technological solutions while prioritizing community engagement and responsiveness to underserved populations’ specific needs and preferences [18]. Utilizing feedback from intended users and understanding their technology usage and behaviors can mitigate the risk of perpetuating further inequalities [19-21]. Thus, the primary objective of this study is to develop and evaluate a novel digital health tool designed to enhance DR screening rates and health education through a community-driven design. By integrating community insights into every stage of the development process, this project aims to bridge the gap between evidence-based screening practices and the actual utilization of these services among those most at risk.

Findings from the 2005 to 2008 National Health and Nutrition Examination Survey showed that 10.6% of their US cohort aged ≥40 years, representing an estimated 9.8 million individuals nationally, were unaware that they had DR [22]. Through a community-led research approach, this project will engage community members directly to identify the barriers and facilitators of DR screening and co-design a digital tool that is accessible, user-friendly, and culturally relevant. The anticipated outcome is a scalable digital health solution that not only increases DR screening rates but also serves as a model for addressing health care disparities in other domains.

## Methods

### Study Design, Study Setting, and Participants

**Figure 1:**
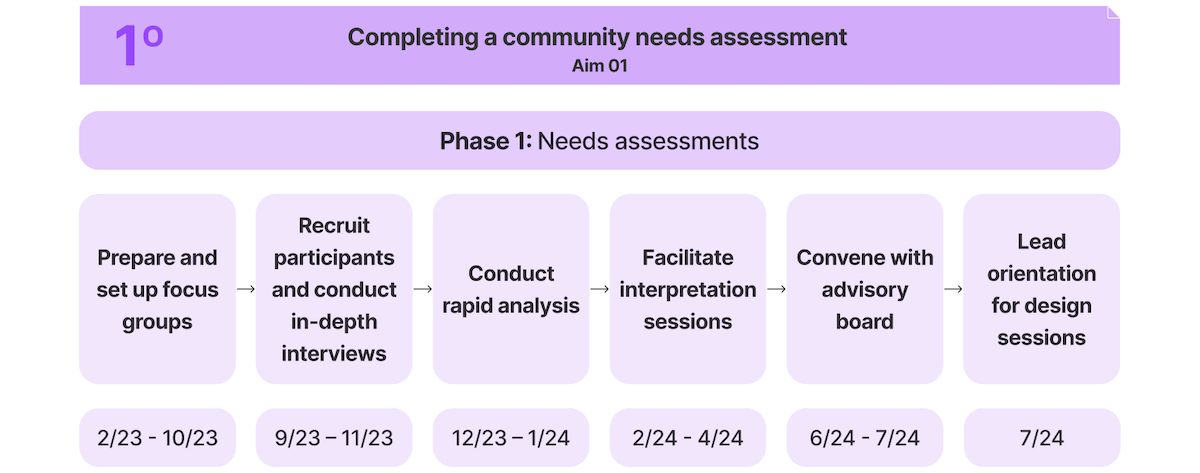
Overview and steps for aim 1 of digital health tool creation.

**Figure 2:**
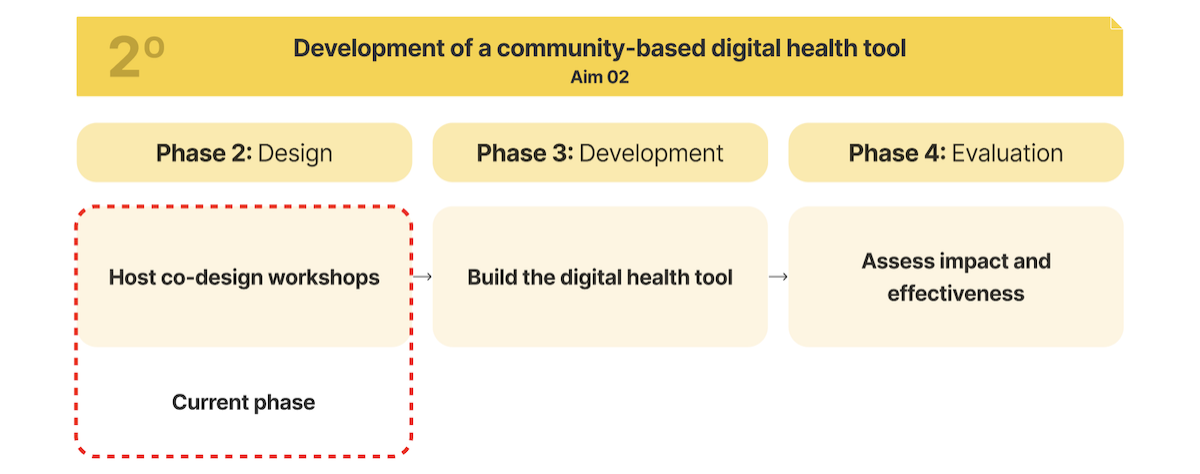
Overview and steps for aim 2 of digital health tool creation.

This protocol has 2 specific aims: (1) to complete a comprehensive needs assessment and (2) to develop a community-responsive digital health tool to increase access to DR screening in high-risk populations and optimize DR education. There will be 4 phases that encompass the 2 aims of the protocol: needs assessment, design, development, and evaluation (Figures 1 and 2). This protocol has been initiated, and we have completed our first aim. We are currently working on our second aim. We recruited individuals with diabetes mellitus from the Greater New Haven, Connecticut area. Results from a study by Guest et al [23] revealed that more than 80% of all themes are discoverable within 2-3 focus groups, and 90% of themes could be discovered within 3-6 focus groups. To ensure rich discussion and engagement, we planned to conduct 4 focus groups with 5-8 participants each session, as smaller groups facilitate deeper participant interaction while still allowing for diverse perspectives.

This research was conducted with the Sight-Saving Engagement and Evaluation in New Haven (SEEN) Lab at the Yale School of Medicine and the Consumer Health Informatics Lab (CHIL) at Yale School of Public Health. The SEEN Lab combines quantitative and qualitative research methods to address health disparities in DR by designing sustainable community-engaged solutions toward equity. CHIL engages in cutting-edge research, focusing on inclusive design and usability testing of consumer health products for diverse populations.

We developed this study in collaboration with the community-engaged research steering committee, a longstanding academic-community partnership. Members of this group provided insight into community-level needs and individual capacities. We convened an advisory board that includes local stakeholders, health care providers, and community leaders to provide insights into the community needs and to guide the research process, ensuring the tool’s development is grounded in real-world contexts. At every phase of this study, we will convene to disseminate findings to the board and to receive guidance on our design and methods in order to continuously address specific community needs and contexts. Each advisory board member will receive a US $50 gift card per advisory board meeting attended.

### Aim 1: Completing a Community Needs Assessment

The first aim of the protocol focuses on conducting a community needs assessment.

#### Phase 1: Needs Assessment

##### Research Design and Data

For our digital health tool, we used 4 theoretical frameworks to help formulate a community needs assessment. The Equity-focused Dissemination and Implementation framework, which provides a foundation for the operationalization of widespread health equity research implementation, served as the overarching guidance for our study’s development [24]. The Consolidated Framework for Implementation Research informed our study’s methodological structure to convene an advisory board, conduct focus groups, and subsequently analyze the focus group transcripts, as this framework gives guidance on organizing implementation [25,26]. The Four Is of Health Equity provide strategies on how to reduce health disparities and promote health equity [27]. We used this framework to steer our study’s objective to focus on identifying health disparities and implementing evidence-based interventions to reduce disparities. The COREQ (Consolidated Criteria for Reporting Qualitative Research) checklist provided a guided reflection of the study upon submission of the manuscript. Lastly, the Reach, Effectiveness, Adoption, Implementation, and Maintenance (RE-AIM) framework informed the metrics the research team will use to evaluate the design process of the digital health tool [28].

##### Recruitment and Interviewing of Individuals Living With Diabetes

Research team members from the SEEN Lab (JF, TC, AF) and CHIL (BA, MK) recruited participants from the Yale Eye Center, primary care offices, and community settings such as restaurants and libraries. We engaged directly with the community through connections and community networks across Greater New Haven community clinics, faith-based organizations, and the Yale New Haven Hospital network [23]. To be eligible for the focus group, participants needed to be at least 18 years old, have a diagnosis of type 1 or type 2 diabetes mellitus, speak English fluently, and be able to attend the interview in person. Although the ability to speak English fluently restricted the inclusivity of our cohort, it was necessary to make this restriction to efficiently design the first prototype of the tool. Future iterations of the tool will account for non-English speakers. Each participant received a US $50 gift card for their participation.

##### Data Collection

The research team (AF, JF, TC, BA, KHN, TM, MS, QY) developed an interview guide in collaboration with the advisory board (Multimedia Appendix 1). The interview guide includes questions about the participants’ experiences with diabetes, their eye health awareness, their technology usage, and their preferences for features in a digital tool to learn about eye health and screening. We collected data from October to November 2023. Semistructured focus group interviews were conducted to gathered qualitative data on participant experiences with diabetes management, awareness of DR, and perceptions of existing health technologies. One member from the research team (AF) served as the moderator, and the remainder served as notetakers (BA, MK, JF, TC, KHN, VHK). The focus groups were audio-recorded, professionally transcribed, and analyzed [24,25]. Participants provided informed consent prior to the interview, and identifying information was removed from the transcript to preserve confidentiality.

##### Data Analysis

The analytic team comprised of members from the SEEN Lab and CHIL (TC, JF, BA, AF, MK, AG, SSA, AL, KHN, TM). Following each focus group interview, the research team conducted the rapid qualitative analysis method to efficiently process data and facilitate interpretation sessions [26,29]. This method allowed the team to systematically collect and analyze participants’ thoughts and experiences within a shorter timeframe [30]. Before the first focus group session, the analytical team developed a structured template (Multimedia Appendix 2) to organize key insights, including the research team members’ initial impressions, detailed accounts of participants’ experiences with diabetes care and the use of technology in health care and eye care, and the team member’s’ summative thoughts on each interview. The research team made minor adjustments to the template as needed to gather more information about eye care experiences. Next, the research team engaged in a series of interpretation sessions with affinity mapping, which is a collaborative process to thematically analyze qualitative data [31]. To keep the information organized, the information was compiled using Miro [32]—an online visual collaboration tool that uses digital sticky notes to help teams organize and synthesize their thoughts. These sessions were moderated by a facilitator (TM). The objective of these sessions was to analyze all the data, which included the original transcripts, summary documents from the rapid review, and notes from each focus group, to systematically code the interviews, categorize responses into emerging themes, and prioritize features. Over multiple sessions, these sticky notes were reorganized to create thematic categories, which will be used to create draft models for the design of the digital health tool [31].

### Aim 2: Development of a Community-Based Digital Health Tool

The second aim consists of 3 phases: design, development, and evaluation of the digital health tool for DR prevention.

#### Phase 1: Design

The design phase leverages insights from the focus groups in the needs assessment phase to cocreate the digital health tool with community members. Using the list of app features and content generated from focus group analysis, the research team created detailed user journey maps to visualize different user experiences as the participants, advisory board members, and other stakeholders interact with the tool—identifying pain points and opportunities to enhance user engagement. First, the research team will collaborate with the advisory board to develop culturally tailored health education content for the tool, including engaging and informative materials on DR, its risk factors, and the importance of regular screening. Then, the research team will develop low-fidelity wireframes and interactive prototypes. These prototypes will be tested and refined through usability testing sessions with community members to ensure that the tool is intuitive and user-friendly.

#### Phase 2: Development and Usability Testing

The next phase consists of building the digital health tool, which will involve translating design prototypes into a functional digital health tool. The research team will collaborate with designers and app developers to build the tool, ensuring it is accessible on various devices and integrates seamlessly with existing health care systems. Lastly, the research team will conduct a usability testing phase with a select group of intended users from the target population. The RE-AIM framework will guide the research team in choosing participants who are representative of the target population and the internal discussion among the research team regarding the tool, especially whether the tool has effectively improved health care management through keeping appointments, encouraging discussion about diabetes, and empowering users to make more informed choices in diet and exercise. The adoption criterion of RE-AIM will also encourage reflection on whether the tool is serving its target population directly. The team will summarize usability walk-through sessions qualitatively. Tobii eye-tracking software will be used to produce heat maps that identify the focus of the participants’ attention on screens to inform user interface design. We will administer the Questionnaire for User Interface Satisfaction and analyze and summarize the open-ended portion. Areas that require optimization will be discussed with the design team, and well-designed areas will be highlighted. Efficiency will be measured by the time required to complete a series of tasks within the app prototype and the number of taps needed to complete each task. We will obtain information on user satisfaction with the app from the metrics calculated from the Questionnaire for User Interface Satisfaction. Statistical analysis will be conducted using SPSS (version 26; IBM Corp). The results of usability testing will inform the app design and help refine the features of the tool through iterative design. Next, we will evaluate the impact of the tool.

#### Phase 3: Evaluation

The final phase, evaluation, will focus on assessing the tool’s impact and effectiveness in increasing DR screening rates. We will implement a pilot study to evaluate the tool in a real-world setting by using the RE-AIM framework to assess its reach, effectiveness, adoption, implementation, and maintenance. Feedback from our intended users will be gathered during the pilot. The feedback will be used to identify and resolve problems, improve usability, and refine tool features before the full launch. We will collect quantitative and qualitative data from users—tracking screening rates, user engagement metrics, and participant feedback.

Lastly, we will conduct iterative usability testing sessions with a diverse group of end users to continuously improve the tool’s interface and functionality. The participants will be adults recruited from local community organizations such as the Dixwell Congregational Church, a historically Black church, with a congregation that spans the ages from children to older adults (65 years and older). Insights from these sessions will inform ongoing adjustments to enhance user experience and ensure the tool meets the community’s needs.

### Ethical Considerations

This study received approval from the Yale School of Medicine Institutional Review Board (IRB # 2000034710). All research procedures were followed in accordance with the Yale School of Medicine institutional review board and the World Medical Association’s Declaration of Helsinki. All participants provided informed consent and were given the opportunity to opt out at any time during the study. All data pertaining to the results of focus groups and related subsequent analyses were deidentified to maintain privacy and confidentiality, and participant-protected health information was stored on an encrypted Yale University information technology department–managed SharePoint server with access restricted to authorized research team members. No images in this manuscript contain participant-identifiable information. Participants received a US $50 gift card for their participation, and advisory board members also received a US $50 gift card for their participation.

## Results

As of December 2023, we completed 4 focus groups with 19 participants. Data from the interviews were used by the research team to conduct descriptive rapid analysis of each focus group session transcript and categorize common themes: the need for enhanced diabetes knowledge, diet-related guidance, informal support through peer connections, integration with electronic health records, and mental health support needed for individuals living with diabetes. The research team then conducted interpretation sessions from February 2024 to April 2024 to efficiently process data, identify pain points, and share findings with the broader study team to start the design process. The team identified features that address the themes of needs expressed by the participants interviewed. The features included a social media component that allows “following” and “liking” posts to create a supportive community, a curated library of recipes for nutritional guidance for people with diabetes, customizable home screens and health metrics like body mass index calculations, integration of functions and data within the MyChart app, appointment reminders, and a curated library of sources of information that served both as a reference for the user and as the training data for a large language model to answer the user’s questions reliably. As of April 2025, the design sprint has been completed, with low and high-fidelity wireframes incorporating the planned features. The next step is to plan for the app’s development and implementation.

## Discussion

### Overview

In the United States, approximately 9.6 million individuals are diagnosed with DR [27]. Unfortunately, many living with DR face barriers to screening and treatment [4,5,7]. DR is a leading cause of blindness, which is preventable and often treatable when patients are empowered to receive timely screening and treatment [1,28,33]. Advancements in technology, such as personal digital health libraries, have helped increase patient engagement and provide the general population with instant access to medical records, including diagnoses and treatment options. Yet, communities of color, Latine, and individuals with lower income are diagnosed and treated less often, experience worse outcomes, and generally engage less with DR care [9-13].

### Anticipated Findings

We anticipate that the patient community with DR needs a culturally responsive digital health tool that prioritizes the communities it serves. Valuable insights from the first phase of the study will enable our development of a tool that aligns with the vision held by the communities it serves. Our community-based approach not only lifts the voices of participants from communities, which often go unheard in health care research, but will also empower them through an educational component, including connecting the participants to resources to help them manage DR.

The culturally responsive digital health tool will include 3 components: a risk calculator, educational features, and self-scheduling software. The risk calculator will help assist providers, especially eye care providers, in clinical decision-making to identify if patients are at a high risk of certain ophthalmic diseases [34]. The educational features will include a large library of curated sources and information, which will also serve to train an artificial intelligence chatbot that will be able to answer queries for users. The self-scheduling software tool will be able to assist with health care navigation by providing appointment reminders and suggestions on which medical practitioners are most recommended by patients.

We will co-design the tool with community partners and continue by using coded interview and user experience data as a guide to iteratively design and evaluate the efficacy of our digital health tool as it is developed. We expect the digital health tool to be well received by and engaging for the Black, Latine, and lower-income communities. We will adhere to the RE-AIM framework to guide the dissemination of our tool, starting with local communities.

### Study Limitations

This study will provide valuable information to help create a digital health tool, however several anticipated limitations can and have appeared in this study. In using focus groups as a methodological approach, there are some limitations that can arise. Some challenges that can arise include recruitment of participants, group dynamics, group composition, sample size, dynamics, and logistic barriers. When designing focus group sessions, a moderate sample size and number of sessions allow for a diverse range of participant experiences and perspectives, ensuring meaningful insights [35]. Although literature suggests a minimum of 4 focus groups for qualitative research, sometimes there is potential for a limited sample size due to various circumstances such as accessibility due to timing, transportation, and availability [36,37]. Despite the research team’s best efforts, participant availability and logistical barriers impacted attendance. Some focus groups had higher attendance than others. The research team recognized this possibility and scheduled multiple sessions in advance, providing convenient weekday and evening sessions, and were flexible to increasing the number of focus group sessions, if needed, to ensure data saturation [35]. Although we could not provide virtual participation, that may be a possibility for future focus group sessions that may allow more accessibility for participants. We also acknowledge that during the recruitment period, we were only able to enroll participants fluent in English. As a result, some individuals were excluded, as proficiency in English was necessary for participation. Future studies could address this limitation by incorporating focus groups with non-English speakers (eg, Spanish only) participants to ensure their perspectives are represented, particularly as they are part of the racial and ethnic minoritized populations.

One inherent limitation of focus group research is selection bias, as participants may not fully represent the broader population. Instead, they tend to provide a snapshot of perspectives from those who chose to participate [38]. The research team aimed to recruit participants with relevant experiences to provide meaningful insights [35]. We carefully considered group composition in terms of number, gender, and age distribution [39,40]. Our focus was to provide a diverse range of perspectives within each focus group. Although we aimed to recruit a diverse sample, we recognize that certain demographic groups may have been underrepresented.

Given the study’s setting within a smaller urban community, there was a possibility that participants knew or were familiar with one another. During the focus group sessions, we emphasized confidentiality at the beginning of each session and ensured that facilitators guided discussions in a way that maintained privacy and encouraged broad, nonidentifying contributions.

Another potential limitation we anticipated was participants’ cultural mistrust, which could influence participants’ willingness to share personal experiences [41]. To foster a comfortable and trusting environment, we ensured that the moderators, facilitators, and/or notetakers were members of the same cultural or community group as the participants. This approach aimed to build rapport and encourage open discussion while minimizing reluctance to disclose information.

Despite these limitations, we believe our approach allowed us to gather meaningful insights that contribute to our study objectives.

### Conclusion

Despite the existence of effective treatments for DR, historically marginalized populations experience poorer health outcomes, including irreversible vision loss. Personal health information technologies have focused on developing patient portals that facilitate communication between patients and providers and enable control of information by the patients themselves. However, their *one-size-fits-all* approach leaves behind communities of color, Latine, and lower-income communities. Our community-based approach empowers the creation of a culturally responsive digital health tool that will engage more of the patient population with DR.

## Appendix

Multimedia Appendix 1 Focus group topic guide.

Multimedia Appendix 2 Diabetic retinopathy digital health tool focus group interview summary template.

Multimedia Appendix 3 COREQ checklist.

## Abbreviations

CHIL: Consumer Health Informatics Lab
COREQ: Consolidated Criteria for Reporting Qualitative Research
DR: diabetic retinopathy
RE-AIM: Reach, Effectiveness, Adoption, Implementation, and Maintenance
SEEN: Sight-Saving Engagement and Evaluation in New Haven
