# Supplementary material for: Digital Health Tool for Preventing Blindness From Diabetic Retinopathy: Protocol for a Qualitative Study
Source: JMIR Res Protoc. 2025 Nov 12;14:e65894. doi: 10.2196/65894 (PMC12658399; doi:10.2196/65894)
Supplement: Multimedia Appendix 1 [file resprot_v14i1e65894_app1.docx]

## Appendix 1:

Introduction

*Good [morning/afternoon/evening]! Thank you for agreeing to participate in the focus group today. My name is [your name], and I am a [your role] for the [name of study]. I would also like to introduce [notetaker name], who will be helping me today by taking notes for our session.*

*The purpose of this focus group is to understand what the community needs are about diabetes and eye care. The information learned in this focus group will be used to inform the design of a mobile app or tool created to learn about eye health and how to get screened for various eye problems. We are very interested to hear about your experience living with diabetes, knowledge about diabetic retinopathy, and ideas about developing a digital health tool. There are just a few things I would like to mention about our session today…*

- *The information you share is completely confidential, and we will not associate your name with anything you say in the focus group.*
- *No questions will be directed to you individually, but instead will be posed to the group. You may choose to respond or not respond at any point during the discussion.*
- *We would like to record the focus groups so that we can make sure to capture the thoughts, opinions, and ideas we hear from the group. You will not be identified in any report or publication of this study or its results. Your name will not appear on any transcripts; instead, you will be given a study ID number. The audio recording will be destroyed as soon as it is transcribed.*
- *We understand how important it is that your information is kept private and confidential. Therefore, we ask that comments made during the focus group session, not be repeated outside of the group by any participants.*
- *Your participation is voluntary, and you may refuse to answer any question or withdraw from the study at any time.*
- *If you have any questions about the study after you leave this session, you can contact me or the lead researcher using the contact information listed in your consent form.*

*Are there any questions before we get started?*

*Okay, let’s start with going around the table and introducing ourselves. Can you say your name before we begin the discussion?*

Let’s start the discussion by talking about how your experience living with diabetes.

Behavioral:

1. How do you typically manage your diabetes on a daily basis, including aspects like blood sugar control, diet and exercise, doctor’s appointments, and medications?
2. How does the influence of friends and family, or the customs and traditions in your community shape your experiences in managing diabetes?
3. What challenges or difficulties have you encountered when trying to receive your DM doctor's appointments?
   1. When was your last full dilated eye exam?
4. Could you provide additional details about the people or resources that are part of your support network for managing your diabetes? This might include family members, friends, healthcare professionals, or any specific programs or tools you use to help with your diabetes management.

Questions specific about the eye:

1. At the time that you were diagnosed with diabetes, what, if anything, were you told about eye care?
2. How did you learn that diabetes can affect your eyes?

For the rest of our session, we will talk about your use of technology, and what information and features you would like in the mobile app or tool to learn about eye health and how to get screened for various eye problems.

Tech

- - 1. Do you currently use any apps or tools for your diabetic care? *Example, if needed: for blood sugar management*
       1. Which apps or tools do you use?
       2. Which features do you like most?
       3. Which features do you like least?

2. What topics would you like to see addressed in the app or tool?

a. Are there any topics that you think would be particularly helpful for people living with diabetes?

3. If you could design an app or tool to support people with managing their diabetes, what kinds of features would it have?

a. Are there any specific activities you would like to be able to complete in the app or tool?

b. What types of notifications would be helpful?

c. How often would you like to receive them?

- - 1. What type of support or resources would be helpful to have information about in the app or tool?
    2. What, if anything, would make you want to use it regularly?
    3. What would get in the way of you using it regularly?
    4. Would you have any concerns about using the app or tool?
    5. If you were designing the app or tool, how would you get the word out about the app or tool?
    6. How do you access technology?
       1. Do you have a cell phone?
       2. Do you have a computer at home?
       3. How often do you use the internet?

Wrap-up Questions:

- - 1. Is there anything you want to go back and address that you did not get the chance to, or that you thought of later?
    2. Is there something you would like to say that we did not ask about specifically?
    3. (To notetakers) Is there anything else you think we should clarify or discuss further?
