# Supplementary material for: Digital Health Tool for Preventing Blindness From Diabetic Retinopathy: Protocol for a Qualitative Study
Source: JMIR Res Protoc. 2025 Nov 12;14:e65894. doi: 10.2196/65894 (PMC12658399; doi:10.2196/65894)
Supplement: Multimedia Appendix 2 [file resprot_v14i1e65894_app2.docx]

## Appendix 2:

# Diabetic Retinopathy Digital Health Tool Focus Group Interview Summary Template

**Analyst:**

**Review date:**

**Focus Group Number:**

# Directions

1. Read the interview transcript cleanly. You want to give yourself a fresh take on the data.
2. Write notes and comments to complete Section 1. Your notes can be bullets, phrases, or full sentences – whatever works for you. If you reference a specific statement please include the quote and participant ID.
3. Review Section 2. Then, read the interview transcript a second time, keeping the questions from Section 2 in mind.
4. Write notes and comments that complete Section 2. Again, use whatever writing format works for you. Include standout or relevant quotes whenever possible, either as full quotes or line numbers. *Note: It's ok to point out when focus group members disagree too (in case you have groups where there isn't an overall feeling).*
5. Review Section 3. Then, read the interview transcript a second time, keeping the questions from Section 3 in mind.
6. Write notes and comments that complete Section 3. Again, use whatever writing format works for you. Include standout or relevant quotes whenever possible, either as full quotes or line numbers. *Note: It's ok to point out when focus group members disagree too (in case you have groups where there isn't an overall feeling).*
7. Review Section 4. Write notes and comments that complete Section 4 (and review the transcript again if needed). Again, you can write in any format that works for you.
8. Review all four sections. Revise and refine any notes that may have changed or shifted throughout your readings.
9. Email your summary template to ______ ahead of the discussion meeting. Make sure you also have easy access to it during the analysis meetings.
10. Thank you!

### Section 1: First Impressions

1. What are your initial impressions after reviewing this transcript?
2. What, if anything, surprised you?

### Section 2: Diabetes Care

1. **Overall, what are the group’s attitudes towards diabetes?**
2. **In the past (ie, since diagnosis), when they were told they have diabetes, did they understand what it meant?**
   1. **Did they understand the symptoms, options for treatment, and side effects of medications that were prescribed? Please add details on what was understood or not understood.**
   2. **What information would they like to have known?**
3. **What health services and resources (eg, diet/lifestyle changes, medications, insulin pump, blood sugar monitoring) have they used?**
   1. **Did they feel that they were helpful?**
4. **What helps them manage their doctors appointments and medications?**
5. **What challenges/barriers make attending appointments or adhering to medications difficult?**
6. **What support systems (influence of friends and family) do they have in place that help their diabetes?**
7. **Were there any resources or health services they wished they had access to help with their diabetes?**
8. **Were there any commonalities with how people were informed about eye care and diabetes?**
9. **Overall, what is the group’s attitude towards diabetes eye care?**
   1. **Were people aware of the benefit of regular eye exams?**
   2. **Did people regularly receive eye exams?**

### Section 3: Tech Use

1. **What experiences did they have with technology for healthcare/diabetes?**
   1. **Do people track their blood sugars through an app?**
   2. **Do people track their steps/activity levels through a smartwatch?**
   3. **What features do they like the most?**
   4. **What features do they like the least?**
2. **Would they want to access health information and resources (blood sugar levels, healthy diet information, exercises, doctors contact info) on a phone/app?**
3. **What do they feel should be included in the mobile app to support diabetes care**
   1. **What topics would be most useful to address? (education, recipes…)**
   2. **What features would be most useful to include? (Doctors contact info, blood sugar monitoring, notifications…)**
4. **Did they have any concerns about using the app?**
5. **What would make them more likely to use the app?**
6. **What would make them less likely to use the app?**
7. **What did they recommend to get the word out about the app?**
8. **How do people generally interact with the internet/technology?**

### Section 4: Summary Thoughts

1. What are your main takeaways from this transcript?
2. What quotes from this transcript stand out to you?
3. If you were coding this transcript line-by-line, what codes would you use?
4. Other comments or reflections that come to mind about this transcript?
