# Supplementary material for: Digital Health Tool for Preventing Blindness From Diabetic Retinopathy: Protocol for a Qualitative Study
Source: JMIR Res Protoc. 2025 Nov 12;14:e65894. doi: 10.2196/65894 (PMC12658399; doi:10.2196/65894)
Supplement: Multimedia Appendix 3 [file resprot_v14i1e65894_app3.docx]

# Appendix 3. COREQ (COnsolidated criteria for REporting Qualitative research) Checklist

A checklist of items that should be included in reports of qualitative research. You must report the page number in your manuscript where you consider each of the items listed in this checklist. If you have not included this information, either revise your manuscript accordingly before submitting or note N/A.

| **Topic** | **Item No.** | **Guide Questions/Description** | **Reported on Page No.** |
| --- | --- | --- | --- |
| **Domain 1: Research team and reflexivity** |  |  |  |
| *Personal characteristics* |  |  |  |
| Interviewer/facilitator | 1 | Which author/s conducted the interview or focus group? | AF, BA, MK, JF, TC, KN, TM |
| Credentials | 2 | What were the researcher’s credentials? E.g. PhD, MD | Researcher (credentials): AF (MD), BA (MD), MK (MS), JF (BS), TC (MD), KN (MD, MBA, MHS) |
| Occupation | 3 | What was their occupation at the time of the study? | AF, BA, JF, MK, and TC were medical students. KN was the principal investigator and study lead. TM was the principal investigator of the CHIL Lab, working under KN. |
| Gender | 4 | Was the researcher male or female? | AF, BA, MK, JF, TM, and KN are females. TC and VR are males. |
| Experience and training | 5 | What experience or training did the researcher have? | KN has extensive training and experience in qualitative, participatory research, particularly with marginalized communities for health disparities, as the principal investigator, and was trained as a retina surgeon. TM has extensive experience and training in participatory research to create culturally sensitive digital tools. AF, BA, JF, MK, and TC were medical students with experience in qualitative research. |
| *Relationship with participants* |  |  |  |
| Relationship established | 6 | Was a relationship established prior to study commencement? | Mostly no, the participants were never contacted prior to the study, except for one participant who was a patient in KN’s clinic. |
| Participant knowledge of the interviewer | 7 | What did the participants know about the researcher? e.g. personal goals, reasons for doing the research | The participants were aware that the interviewers were graduate students involved in the research. Additionally, Dr. Nwanyanwu was a healthcare provider for one of them. |
| Interviewer characteristics | 8 | What characteristics were reported about the inter viewer/facilitator? e.g. Bias, assumptions, reasons and interests in the research topic | They knew that Dr. Nwanyanwu was a healthcare provider, which may have shaped perceptions of the team’s interest and credibility in the research topic. |
| **Domain 2: Study design** |  |  |  |
| *Theoretical framework* |  |  |  |
| Methodological orientation and Theory | 9 | What methodological orientation was stated to underpin the study? e.g.  grounded theory, discourse analysis, ethnography, phenomenology, content analysis | (5) The study was based on 4 pre-established and widely recognized frameworks from grounded theory, used in health equity research: (1) the Equity-focused Dissemination and Implementation (EQ-DI) framework, (2) the Consolidated Framework for Implementation Research (CFIR), (3) the Four I’s of Health Equity, and (4) the Reach, Effectiveness, Adoption, Implementation, and Maintenance (RE-AIM) framework. |
| *Participant selection* |  |  |  |
| Sampling | 10 | How were participants selected? e.g. purposive, convenience, consecutive, snowball | Participants were selected using a combination of purposive, convenience, and snowball sampling methods. Flyers were posted in clinical settings where individuals were likely to meet the study’s eligibility criteria (purposive sampling). Some participants were contacted directly based on prior relationships or accessibility (convenience sampling). Additionally, in a few cases, enrolled participants referred or brought others to the study (snowball sampling). |
| Method of approach | 11 | How were participants approached? e.g. face-to-face, telephone, mail, email | Participants were primarily approached via telephone and email. Some individuals initiated contact themselves by scanning QR codes on recruitment flyers. In one instance, a participant was approached face-to-face during a clinical appointment with Dr. Nwanyanwu. Research team members from the SEEN Lab (JF, TC, AF) and CHIL (BA, MK) recruited participants from the Yale Eye Center, primary care offices, and community settings such as restaurants and libraries. The team also engaged directly with the community through established relationships and networks across Greater New Haven, including community clinics, faith-based organizations (Dixwell Congregational Church), and the Yale New Haven Hospital (YNHH) network. |
| Sample size | 12 | How many participants were in the study? | (8) 19 individuals |
| Non-participation | 13 | How many people refused to participate or dropped out? Reasons? | A total of 24 individuals were initially recruited. Several individuals were ultimately unable to participate due to scheduling conflicts or other time constraints. In some cases, participants did not respond to follow-up communications or failed to attend the scheduled session without providing a reason. |
| *Setting* |  |  |  |
| Setting of data collection | 14 | Where was the data collected? e.g. home, clinic, workplace | Data were collected in person at two locations: the Dixwell Q House (217 Dixwell Ave, New Haven, CT) and a local café that was opened privately for the first focus group session. The café was not open to the public at the time and was reserved exclusively for the study. Subsequent sessions were held at the Q House. |
| Presence of nonparticipants | 15 | Was anyone else present besides the participants and researchers? | (5) No. All sessions were conducted in private settings with only participants and research team members present. At the Q House, discussions took place in a soundproof room to ensure privacy and confidentiality. The café used for the first session was also closed to the public and reserved exclusively for the focus group. |
| Description of sample | 16 | What are the important characteristics of the sample? e.g. demographic data, date | (Abstract) We recruited a diverse cohort of participants. The sample was 68% female, 47% identified as Black, 26% as Hispanic, and 11% as Indigenous. Nearly half of participants (48%) had completed some college education. Over 80% reported having access to smart devices. Participants also self-reported a mean hemoglobin A1c of 6.77 (SD = 1.93), reflecting a range of glycemic control across the sample. |
| *Data collection* |  |  |  |
| Interview guide | 17 | Were questions, prompts, guides provided by the authors? Was it pilot tested? | (5) The interview guide (Appendix 1) was derived after consultation with the Advisory Board.I |
| Repeat interviews | 18 | Were repeat inter views carried out? If yes, how many? | No repeat interviews were conducted. Four separate focus groups were held, each with a different set of participants. |
| Audio/visual recording | 19 | Did the research use audio or visual recording to collect the data? | (4, 5) Only audio recording was used for the focus groups. These interviews were recorded, then an independent transcription service vendor transcribed them, and the research team coded them. |
| Field notes | 20 | Were field notes made during and/or after the inter view or focus group? | Yes, field notes were taken both during and after the focus group sessions. These notes captured the research team’s impressions and contextual observations about each session. Dr. Terika McCall (TM) guided the subsequent rapid analysis sessions, during which the field notes were reviewed and incorporated into the analytic process. |
| Duration | 21 | What was the duration of the inter views or focus group? | Each focus group session lasted approximately 90 minutes. The first 30 minutes were dedicated to dinner and a warm welcome, followed by a 60-minute moderated discussion. |
| Data saturation | 22 | Was data saturation discussed? | (4) Yes, data saturation was considered during the study design. Based on prior research by Guest et al., which demonstrated that approximately 80% of themes emerge within two to three focus groups and 90% within three to six groups, we anticipated that conducting three to five focus groups would be sufficient to reach thematic saturation. To support rich discussion and ensure engagement, each focus group was designed to include 5 to 8 participants, a size that balances depth of interaction with diversity of perspectives. |
| Transcripts returned | 23 | Were transcripts returned to participants for comment and/or | No, transcripts were never returned to the participants, only ever reviewed by the research team. |
| **Topic** | **Item No.** | **Guide Questions/Description** | **Reported on Page No.** |
|  |  | correction? | See above |
| **Domain 3: analysis and findings** |  |  |  |
| *Data analysis* |  |  |  |
| Number of data coders | 24 | How many data coders coded the data? | (6) The analytic team comprised of 5 coding members from the SEEN Lab and CHIL (TC, JF, BA, AF, MK). |
| Description of the coding tree | 25 | Did authors provide a description of the coding tree? | Yes, coded themes and corresponding emerging broad themes were identified.However, no formalization of the hierarchy into a coding tree was generated. |
| Derivation of themes | 26 | Were themes identified in advance or derived from the data? | Themes were derived from the data through team-based interpretation sessions. The research team did not impose predefined themes; instead, themes emerged organically through discussion and analysis of participants’ responses. |
| Software | 27 | What software, if applicable, was used to manage the data? | (6) We used Miro, an online collaborative platform, to facilitate affinity mapping by organizing digital sticky notes into thematic clusters during the analysis process. Microsoft Office tools were used for documenting and organizing transcripts and notes. Additionally, Box was used for the secure, encrypted storage of sensitive data to ensure confidentiality and compliance with institutional data protection standards. |
| Participant checking | 28 | Did participants provide feedback on the findings? | (6, 7) Participants did not provide immediate feedback on the findings during the initial analysis phase. However, they will be invited to review and comment on the findings during final step of the design phase (Phase 1). At that time, the wireframes with illustrations of prospective features will be presented to the broad local New Haven community for feedback. Additionally, participant feedback will be incorporated in Phases 2 and 3 of the study, which include usability testing, to ensure the tool remains responsive to user needs. |
| *Reporting* |  |  |  |
| Quotations presented | 29 | Were participant quotations presented to illustrate the themes/findings?  Was each quotation identified? e.g. participant number | Yes, participant quotations were used to illustrate key themes and findings. During rapid analysis and affinity mapping, the research team reviewed transcripts and selected quotations that resonated with emerging concepts. Although quotations were not attributed to individual speakers during the initial analysis, the final manuscript includes de-identified excerpts labeled by participant number (e.g., Participant #1) to provide context while maintaining confidentiality. |
| Data and findings consistent | 30 | Was there consistency between the data presented and the findings? | Yes, we ensured consistency between the data presented and the study’s findings. Our use of established implementation and equity frameworks—such as CFIR, RE-AIM, and the Four I’s of Health Equity—helped guide a rigorous and transparent analytic process. These frameworks supported alignment between the data, interpretation, and reported themes, contributing to the overall quality and coherence of the research. |
| Clarity of major themes | 31 | Were major themes clearly presented in the findings? | Yes, the major themes were clearly presented in the findings. The discussion section, in Anticipated Findings, makes reference to the features that were inspired by the themes abstracted from focus groups and rapid analysis. |
| Clarity of minor themes | 32 | Is there a description of diverse cases or discussion of minor themes? | Complete details on the results and codes found and abstracted from the focus groups can be found in another manuscript that exclusively focuses on the affinity mapping process. |

Developed from: Tong A, Sainsbury P, Craig J. Consolidated criteria for reporting qualitative research (COREQ): a 32-item checklist for interviews and focus groups. *International Journal for Quality in Health Care*. 2007. Volume 19, Number 6: pp. 349 – 357

**Once you have completed this checklist, please save a copy and upload it as part of your submission. DO NOT** **include this checklist as part of the main manuscript document. It must be uploaded as a separate file.**
